# Mindfulness-based stress reduction for community-dwelling older adults with subjective cognitive decline (SCD) and mild cognitive impairment (MCI) in primary care: a mixed-methods feasibility randomized control trial

**DOI:** 10.1186/s12875-023-02002-y

**Published:** 2023-02-09

**Authors:** Todd Tran, Catherine Donnelly, Emily Nalder, Tracy Trothen, Marcia Finlayson

**Affiliations:** 1 School of Rehabilitation Therapy, Queen’s University, Louise D. Acton Building, 31 George Street, Kingston, Ontario K7L 3N6 Canada; 2 Clinical Site: Women’s College Hospital, 76 Grenville St., Toronto, Ontario M5S 1B2 Canada; 3 Department of Occupational Science & Occupational Therapy, University of Toronto, 500 University Ave, Toronto, ON M5G 1V7 Canada; 4 Jointly appointed to the School of Rehabilitation Therapy and School of Religion (Theological Hall), Queen’s University, Louise D. Acton Building, 31 George Street, Kingston, Ontario K7L 3N6 Canada

**Keywords:** Mindfulness, Occupational therapy, Subjective cognitive decline, Mild cognitive impairment, Interprofessional primary care, Technology

## Abstract

**Background:**

Primary care is often the first point of contact when community-dwelling older adults experience subjective cognitive decline (SCD) or mild cognitive impairment (MCI). Living with SCD or MCI can be life-altering, resulting in low mood and increased anxiety, further exacerbating cognitive decline. However, there is scant literature on interventions that interprofessional primary care providers can provide to support those living with SCD or MCI. Practicing mindfulness-based stress reduction (MBSR) in an interprofessional primary care setting may support emotional health and well-being for those with cognitive decline, but it has not been studied in an interprofessional primary care context.

**Objectives:**

This study’s primary aim was to determine the feasibility of, and perceived benefits to and satisfaction with, a 9-Week MBSR program delivered in a team-based primary care setting. The secondary aim was to examine the acceptability of using technology (computer tablet and App Insight Timer®) for program delivery and home practice.

**Methods:**

A convergent mixed-methods, single-blind pilot randomized controlled trial (RCT) study design was used. A quantitative strand was used to evaluate the feasibility of the MBSR program. The qualitative strand used a focus group with older adult participants with SCD or MCI. Individual semi-structured interviews with occupational therapists who are qualified-MBSR teachers were conducted to explore the acceptability of using computer tablets for program delivery and home practice.

**Results:**

27 participants were randomized (14 MBSR; 13 Control) with retention rates of 64.3% (9/14 completed ≥6 sessions), true adherence rates of 50% (7/14 met ≥19.5 hrs of home practice), 21.4% attrition rates, and 100% post-intervention follow-up. No participants who used computer tablets at the beginning of the intervention switched to low technology. Older adult participants found the use of computer tablets in the MBSR course acceptable and appreciated the portability of the tablets.

**Conclusions:**

Based on the lower-than-expected rates of recruitment, retention, and adherence, our study, as designed, did not meet the feasibility benchmarks that were set. However, with minor modifications to the design, including changing how participants who drop-out are analyzed, extending recruitment, and adding multiple sites, this intervention would be well suited to further study using a full-scale RCT. However, we found that embedding MBSR in an interprofessional primary care setting was feasible in practice and qualitative data highlighted the satisfaction and perceived benefits based on the intervention. The use of technology was acceptable and portable, as participants utilized their computer tablets consistently until the study’s end. Our study showed that older adults living with SCD or MCI were highly receptive to learning how to use technology, and future group intervention programs in interprofessional primary care settings may also incorporate tablet use.

**Trial registration:**

This study was reviewed and approved by the Research Ethics Board in Toronto, Ontario, Canada (REB# 2017–0056-E); Queen’s University (REB# 6026418) in Kingston, Ontario, Canada, and Clinicaltrials.gov (08/03/2019; NCT03867474).

**Supplementary Information:**

The online version contains supplementary material available at 10.1186/s12875-023-02002-y.

## Background

According to the World Health Organization (WHO, 2018), the world’s population of adults aged 60 years and older is expected to reach 2 billion by 2050. As older adults live longer, the risk of developing neurodegenerative diseases, such as Alzheimer’s disease (AD), increases. A *Canadian Study on Health and Aging* estimated that there are currently 564,000 persons living with dementia, and this number will increase to 937,000 by 2031. Of note, more than 65% are projected to be women (Health Canada, 2016, October).

It is estimated that one-third of community-dwelling older adults will experience cognitive complaints [1]. Experiences of cognitive decline vary, including for individuals with the earliest signs of memory complaint defined as subjective cognitive decline (SCD), a self-reported subtle decline in cognitive performance, without objective impairment on cognitive assessment [2]. The next stage of cognitive dysfunction [3] is mild cognitive impairment (MCI), which is defined as having an impairment in one or more of the cognitive domains relative to appropriate normative data for that individual [4]. The prevalence of MCI is between 15 and 20% in older adults aged ≥ 60 years, and the rate of MCI progressing to dementia, a more severe cognitive impairment, is between 8 to 15% per year [5].

As cognitive performance declines, older adults with SCD or MCI report inefficiency with day-to-day functioning, particularly complex instrumental activities of daily living, such as financial management and shopping [6]. Such individuals may take longer to complete complex tasks and make more errors than in the past [4, 7], while basic activities of daily living such as bathing, dressing and feeding are all intact [8]. This subtle functional decline may result in a general sense of dissatisfaction and discontentment experienced by older adults with respect to their overall functional performance [9]. Furthermore, the emotional aspect of living with SCD or receiving an MCI diagnosis can negatively impact an individual’s emotional health and well-being [10]. As such, it may raise fears of dependency on others, and activity limitation [11], which may result in depressive mood and increased anxiety [10, 12, 13].

The efficacy of pharmacological management to improve concomitant anxiety disorders and depression among older adults living with cognitive impairment is questionable [14–18]. Researchers have found that pharmacological interventions are over-prescribed in older adults, despite the potential risk of drug-induced side effects [17], drug complications [19], and falls [20].

Clinical guidelines for the management of cognitive impairment in primary care prioritize non-pharmacological interventions, which are appropriate to patients’ cognitive and physical capabilities [21]. Therefore, identifying effective non-pharmacological interventions to mitigate psychosocial factors, such as anxiety and low mood, and supporting functional performance with those living with SCD or MCI is warranted [17, 18]. The expanding focus of interventional research in primary care is to evaluate management strategies to reduce symptoms before further irreversible dysfunction has occurred for older adults at risk of developing AD. Ideally, reducing the incidence and prevalence of AD [22] would be crucial.

Primary care teams are gaining increased attention in Canada as potentially being a significant way to help address the complexities of the changing Canadian demographics, including an aging population and an increase in chronic physical and mental health conditions. It has been noted that a holistic approach to care that addresses the well-being and mental health of older adults is imperative [14]. Primary care teams can offer this broader lens through the use of mindfulness-based stress reduction (MBSR), a 9-Week program that was first introduced in 1979 by Jon Kabat-Zinn [23]. MBSR is used to treat a variety of clinical psychiatric diagnoses (e.g. depression, anxiety, perceived stress) [24–26] and to help cope with other clinical illnesses (e.g. HIV, diabetes, cancer) [27–29]. MBSR is widely taught in hospitals, community centres, universities, schools and in private practice. Since its inception, MBSR has demonstrated considerable positive mental health outcomes for the general adult population [30, 31]. More recently, small proof-of-concept and pilot studies have found MBSR to be feasible with older adults with early cognitive deficits such as SCD and MCI [32–34]. Furthermore, there is mounting evidence that mindfulness-based interventions such as MBSR can help community-dwelling older adults living with cognitive dysfunction to self-manage anxiety, depressive symptoms, as well as perceived stress in the context of SCD or MCI, which in turn may support their emotional health and well-being [35–37].

Primary care providers on Family Health Teams (FHTs) are usually the first point of contact when older adults experience cognitive problems such as SCD or MCI [38]. There is an increasing emphasis on these interprofessional primary care teams to support and address the healthcare needs of the aging population. However, there has been limited research examining the benefits of an MBSR program with older adults living with cognitive complaints, and no studies have examined the use of MBSR in an interprofessional primary care setting with this population. Drawing on the expertise and understanding of cognitive problems and their impact on daily functioning, occupational therapists working on primary care teams are well-positioned to address the functional and psychosocial needs of community-dwelling older adults living with SCD or MCI. However, there is currently scant information on the types of health services provided by occupational therapists to older adults with SCD or MCI in primary care settings.

This study’s primary objective was to evaluate the feasibility of an occupational therapist-led MBSR program, for community-dwelling older adults living with SCD or MCI, in primary care. The secondary objective was to evaluate the acceptability of using technology (e.g., computer tablets and App Insight Timer®) as a tool for program delivery and home practice.

## Methods

### Study design

A convergent mixed-methods, single-blind pilot randomized controlled trial (RCT) with quantitative and qualitative strands was used to assess the feasibility of a 9-Week MBSR program, and the acceptability of using computer tablets for program delivery and home practice.

### Ethical considerations

This study was approved, and all methods were performed in accordance with guidelines and regulations issued by the Research Ethics Board in Toronto, Ontario, Canada (REB# 2017–0056-E) and Queen’s University (REB# 6026418) in Kingston, Ontario, Canada.

### Study setting

The study took place between August and October 2019 in one academic FHT in a large urban city in the province of Ontario, Canada. FHTs are a model of interprofessional primary care and were introduced in 2006 in Ontario. The FHT is considered a patient medical home, and approximately 25% of Ontarians receive their primary care from this model of care. Primary care teams consist of family physicians and interprofessional health care providers such as: dieticians, occupational therapists, pharmacists, physiotherapists, and social workers [39], all of whom work together to provide comprehensive primary care. The FHT that collected the data included 36 physicians, two dieticians, one occupational therapist, one physiotherapist, two social workers, and one pharmacist, and it had approximately 18,000 rostered patients.

### Eligibility criteria

This study recruited rostered patients from the FHT with the following inclusion criteria: (i) aged ≥ 60 years; (ii) fluent in English; (iii) living independently in the community (non-assisted living, e.g., retirement or any long-term care facility); and (iv) had a cognitive complaint. SCD was assessed using: (i) Jessen et al.’s (2014) criteria of self-experienced persistent decline in cognitive capacity in comparison with a previously normal status and unrelated to an acute event; and (ii) a Montreal Cognitive Assessment (MoCA) score of ≥24 to ensure objectively they do not have MCI [2]. On the other hand, clinical characterization of MCI was assessed using Petersen et al.’s criteria (2014) of self-reported cognitive complaint, with an objective cognitive impairment on the MoCA with a score of ≤23 [4, 40] or had a confirmed MCI diagnosis noted in their electronic medical record (EMR). Exclusion criteria were: (i) history of prior participation in an MBSR program or having ≥1.5 hrs of other mindfulness practices, including yoga; (ii) currently experiencing significant health issues (e.g., receiving chemotherapy, brain injury) or a psychiatric condition (e.g., significant clinical depression); (iii) active use of alcohol or substance use; (iv) involved in another cognitive or memory program or another research study.

### Recruitment

Participants were recruited within the FHT through posters placed in waiting areas, clinics, and physician consult rooms. Interprofessional primary care providers also recruited potential participants for the study. The recruitment period was approximately four and a half months in duration.

### Treatment allocation and randomization

Once informed consent was obtained from all study participants, they were randomly allocated to either a 9-Week MBSR program or to a 9-Week wait-list control group. A research staff member not involved in the study design prepared the randomization sequence in opaque sealed envelopes to ensure allocation concealment for distribution. All research staff, including the Principal Investigator (PI), were blinded to the randomization list. The wait-list control group received the MBSR intervention post-three-months when the experimental group was completed. Our study protocol has been published previously [41]. All original study data, from focus group interviews to feasibility data such as attendance and field notes, were all kept under lock and key in a secure location within the clinic.

### Intervention/treatment (MBSR) group

All MBSR programs consist of an orientation session followed by eight three-hour weekly sessions. Each session consists of various mindfulness practices such as lying down (body scan), sitting (attention on the breath), and mindful movement (yoga and walking). The curriculum also includes an all-day retreat. In our study, the all-day retreat was originally scheduled for the Saturday of Week-6; however, it was moved to the Wednesday of Week-7, thereby extending the program to 9 weeks. See Table 1. As part of our study, participants were also asked to continue with 30 minutes (6 days a week) home practice after the 9-Week program for another 4 weeks and were followed-up at the end of study at Week-13. See Table 2.

**Table 1:** Outline of the MBSR/intervention

Week	Session (3 hrs duration)	MBSR/Intervention
0	Orientation	Objective of the intervention clarified, dispensing, and demonstrating computer tablets and App Insight Timer. Technological support provided to ensure understanding. Logistics of the MBSR course; formal and informal practices; what to wear/bring; attendance; importance of home practice.
1	1	Introductions in dyads, establishing group norms. Formal practices: mindful eating raisin task, inquiry, break, body scan and inquiry. Home practices were provided – doing the Body scan 6 days out of the week, reading the Upstream and Downstream fable, eating one meal mindfully, and doing the nine-dot exercise.
2	2	Mindful standing movement along with a formal practice of the body scan and inquiry. Home practice will be taken up and discussed. A formal practice of attention on the breath (AOB) will be also introduced along with home practice for next week (e.g., pleasant event calendar, body scan, mindful routine activity and AOB).
3	3	Sitting practice of Attention on the Breath (AOB), along with a formal practice of lying down yoga and with a short body scan and inquiry. Home practice will be taken and discussed regarding the body scan at home, and pleasant event calendar. Home practice for next week will consist of (e.g., unpleasant event calendar, alternating body scan with mindful lying down movement, and self-guided AOB for 5 to 10 mins).
4	4	Standing yoga, with a short 20 mins guided meditation practice and inquiry in dyads, then in a large group, and with taking up the home practice. Taking up unpleasant event calendar and will provide an interactive stress reactivity education session. Home practices will be provided – alternating the Body scan with standing mindful movement 6 days out of the week, and observing stress reactions by noticing (thoughts, moods and behaviour).
5	5	Mindful movement and a sitting practice, with a practice inquiry. Facilitator will ask participants to reflect on the first half of the program with reflective questions as an invitation to recommit to practice for the remainder of the program. Stress reaction versus response was presented in class along with noticing habits and changing response i.e., cow path. Facilitator will introduce S.T.O.P. as a responsive strategy.
6	6	Longer sitting meditation, with a practice inquiry. In dyads, participants will discuss stressful communication, and in large group take up the home practice and discuss intention to cultivate awareness, exploring patterns of communication, and noticing habitual patterns and behaviour (not only in the realm of interpersonal communication, but also in one’s inner life). Facilitator will enact Aikido of styles of communications (aggression, passive, conflict, and assertiveness). Discuss new ways of experimenting with “new behaviour” and ways of engaging interpersonally.
7	All Day Silent Retreat	Traditionally done on a weekend; but moved to the following week. A 6-hour long retreat for to deepen the practice in order to cultivate a sense of presence and attention on the present moment and experience.
8	7	Mindful movement of yoga choices, and a sitting practice with a practice inquiry. Inquiry will be around mindful awareness that can change relationship to stress and increase choice along with integrating mindfulness more fully into our lives can increase resilience. Different Chair Exercise will be introduced and discussion of attachment or aversion, habitual patterns and beginner’s mind. Home practice will be around consumption and developing healthier patterns.
9	8	Reflective practice of the past eight weeks and what participants have learned, the cost or sacrifice made to attend this course. Last practice will be the body scan along with inquiry. Closing ceremony will include the yarn bracelet and giving participants the certificates for course completion. Participants with written and verbal consent will participate in a focus group later in the week.

**Table 2:** Timeframe of measurements for participants in MBSR intervention

Measures taken	Time-1 (T1)									Time-2 (T2)	Time-3 (T3)
	(Orientation)							All-Day		(post-MBSR)	(follow-up)
Item	0-week	1-week	2-week	3-week	4-week	5-week	6-week	7-week	8-week	9-week	13-week
**Quantitative measures**											
Feasibility measures	X	X	X	X	X	X	X	X	X	X	X
**Qualitative measures**											
Focus group (Participants)										**X**	
Interview with MBSR teachers											**X**

Commonly, MBSR is taught by two instructors. However, in this study, the MBSR course was facilitated by four occupational therapists who were qualified MBSR teachers. Two were primary teachers, and two supported participant adherence and addressed any technological issues that arose before or after the program. MBSR teaching experience ranged between six and 12 years, respectively. Three of the four were trained in the United States (University of Massachusetts or Brown University) and one in Toronto, Ontario, Canada.

Unlike standard MBSR programs, we introduced an additional component. Each participant who had access to Wi-Fi received a tablet computer (mini-iPad3) to use the Application (App), Insight Timer® [42]. The App contained guided meditations for daily home practices (30–45 min) for the duration of the study (13-Weeks). Participants were able to access home practice by logging directly onto their tablet and accessing the App. In addition, participants were provided with self-report home practice diary sheets to capture their formal and informal practice.

## Data collection

### Quantitative data

#### Feasibility outcome measures and acceptability of technology

The App was used to collect the frequency of logins and home practice duration and was downloaded weekly onto an Excel spreadsheet by a research assistant at arm’s length from the study for data entry and analysis. Participants who had CD players but without Wi-Fi access, were given CDs to use for their guided home practices. However, we instructed all participants to record their home practices using pen and paper daily logs as a backup if a technology malfunctioned.

As previously described, feasibility outcomes included the following objectives: 1(a) patient recruitment, study retention, intervention adherence, and follow-up rates; and 1(b) acceptability of technology [41]. Data were collected from attendance records, home practice duration and completion, and computer tablet logins; attrition throughout the MBSR program was also tracked.

##### Objectives 1(a): feasibility outcome measures

Recruitment rate: A target recruitment rate to demonstrate feasibility was defined as 30 plus participants between May and August 2019 (4.5 months), or seven people per month, similar to other feasibility studies [43] with an enrollment rate of 70%.
Retention rate: The target retention rate was at least 75–80% of participants completing six or more of the 10 sessions (including orientation and all-day session), for retention to be deemed successful. In addition, there was a follow-up at Week-13 (e.g., tablet and self-reported home diary collection). A withdrawal rate of 20% or less was deemed to be indicative of program feasibility based on other feasibility studies [44].
(i) Adherence: An adherence rate was used for participants using tablets, and a true adherence rate was used for participants using tablets and CDs, thus capturing all program participants (those that chose CDs or computer tablets).

(ii) Adherence rate (tablets only): The adherence rate was deemed adequate if (i) participants completed three logins per week or 39 logins (3 logins per week × 13-weeks) and (ii) formal practice for at least 1.5 hours (90 mins) per week or 19.5 hours (1.5 hours × 13-weeks; 1170 mins) duration of the study, beyond the 9-week intervention.

(iii) True Adherence rate (tablets and CDs): Formal meditation home practice is setting aside time to carry out the guided meditation recordings. Informal meditation practice is bringing awareness within the flow of participants’ day-to-day activities at any time. As such, to capture both formal and informal practices, a true adherence rate was determined by both i.) attendance rate (attending ≥ 6 or more sessions) and ii.) self-reported home practice diary consisting of formal practice and informal practice totaling ≥ 19.5 hrs (or ≥ 1170 mins) during the 13-week study. True adherence also includes formal and informal meditation home practice (in minutes).

4. Duration of home practice: Comparing both tablet data collection of formal home practice and self-reported diary home practice logs to determine level of accuracy.

##### Objectives 1(b): acceptability of technology

Acceptability of using tablet computer as a tool for home practice delivery was determined by: (1) field notes by qualified-MBSR teachers documenting group participation, (2) number of participants that switched from computer tablets to low technology (e.g., CDs) for the homework practices for the duration of the MBSR program and (3) focus groups (T2) examining perceived value and benefits of using technology.

### Qualitative data

#### Qualitative data collection

A focus group was conducted with participants at Week-9, after the last three-hour weekly session, to explore their experience with the MBSR program, and acceptance of technology for program delivery and home practices. The focus group was held at the FHT clinic where the study was conducted. In addition, semi-structured interviews were conducted with the qualified MBSR teachers to explore their experience providing the program. The focus group and interviews were audio-recorded, and transcribed verbatim. Participants were de-identified and given a participant number.

## Data analysis

### Feasibility outcome measures and acceptability of technology

Feasibility data were entered into an Excel spreadsheet and analyzed using descriptive statistics, including mean, rates, and standard deviations.

#### Qualitative analysis

All supportive quotations from the focus group and individual interview transcripts were anonymized and analyzed using thematic analysis described by Braun and Clarke [45]. Qualitative software NVivo (Version 12) was employed to support analysis. Investigators used data source triangulation to collect data from the different sources, such as a focus group and in-depth individual interviews. These interviews allowed for spontaneity, flexibility, and responsiveness by eliciting information regarding their personal experiences and perspectives [46]. The PI, a co-author and a research team member and author ensured trustworthiness by peer debriefing to discuss their independent coding schemes. Generated codes were presented to the research team and stored in a master codebook. An audit trail was kept and maintained to document decision-making pathways, and reasoning of final analysis. These strategies enhanced trustworthiness by ensuring dependability, credibility and transferability [47].

##### Data integration

Data integration and analysis used a narrative approach that described the quantitative, and qualitative results thematically. As such, this narrative approach weaved together the feasibility outcome to the qualitative themes related to satisfaction and perceived benefits of the MBSR program [48].

## Results

There were five participants in the MBSR group that met the MCI inclusion eligibility for our study. For MCI confirmation, two participants in the MBSR group met the criteria for MCI of: (i) MoCA score of ≤23; and (ii) an MCI label by their primary care physician in their EMR. Conversely, another three participants in the MBSR group had a MoCA score of ≤23 but lacked the MCI label in their EMR, in which case Petersen et al.’s MCI criteria was used to assess the participants’ cognitive complaints for MCI to meet the study’s eligibility.

Participants’ characteristics are summarized in Table 3. Control and intervention groups were similar and had no clinically relevant differences.

**Table 3:** Characteristics of 14 participants in the 9-week MBSR program

***Characteristics***	Total (No., % total)	
	**MBSR *n*** **= 14**	**Control *n*** **= 13**
**Age (Mean; SD), years**	71.43 (9.0)	75.31 (9.5)
Range	60–83	62–92
**Sex: Female**	8 (57.1%)	12 (92.3%
**Education Level**		
None	2 (14.3%)	1 (7.7%)
High School	3 (21.4%)	2 (15.4%)
College	0 (0%)	3 (23.1%)
University	9 (64.3%)	7 (53.8%)
**Living Arrangement**		
Alone	4 (28.6%)	8 (61.5%)
With partner	6 (42.9%)	2 (15.4%)
With family/friends	4 (28.6%)	3 (23.1%)
**Marital Status**		
Married	11 (78.6%)	11 (84.6%)
Common-law	1 (7.1%)	2 (15.4%)
Other	2 (14.2%)	0
**Currently Employed**		
Yes (full-time or part-time)	6 (42.9%)	2 (15.3%)
No (retired)	8 (57.1%)	11 (84.6%)
**Household Income**		
< $50 K	8 (57%)	6 (57%)
$51-99 K	2 (14.2%)	5 (38.5%)
> $100 K	4 (28.6%)	2 (15.4%)
**Have Driver’s License**		
Yes	11 (78.6%)	5 (38.5%)
**Currently Driving**		
No	7 (50%)	9 (69.2%)
**Previous Head Injury**		
Yes	5 (35.7%)	5 (38.5%)
**Duration of Physical Activity** **(e.g., walking, etc.)**		
**(Mean; SD) hrs/week**	3.83 (3.21)	6.76 (4.60)
**Meditation Practice (≤ 1.5 hrs/week)**	3 (21.4%)	2 (15.4%)
**Those who own an iPAD**		
Yes	8 (57.1%)	5 (38.5%)
**Have Used an iPAD before**		
Yes	8 (57.1%)	6 (46.2%)
**Experience with iPAD**		
No experience	7 (50%)	9 (69.2%)
1 yr	2 (14.2%)	0 (00.0%)
2 yr	1 (7.1%)	0 (00.0%)
4 yr	0 (00.0%)	2 (15.3%)
5 yr	4 (28.5%)	2 (15.3%)
**Montreal Cognitive Assessment (MoCA)**		
	25.00 (2.45)	25.92 (2.29)
**MCI Diagnosis**		
Yes	2 (14.3%)	2 (15.4%)
No (but MoCA ≤23)	3 (21.4%)	0 (0.00%)
**Geriatric Depression Scale (GDS)**	2.14 (2.68)	2.92 (2.49)

### Feasibility outcome measures and acceptability of technology

#### Objectives 1(a): feasibility outcome measures

1. *Recruitment rates*

**Fig. 1:**
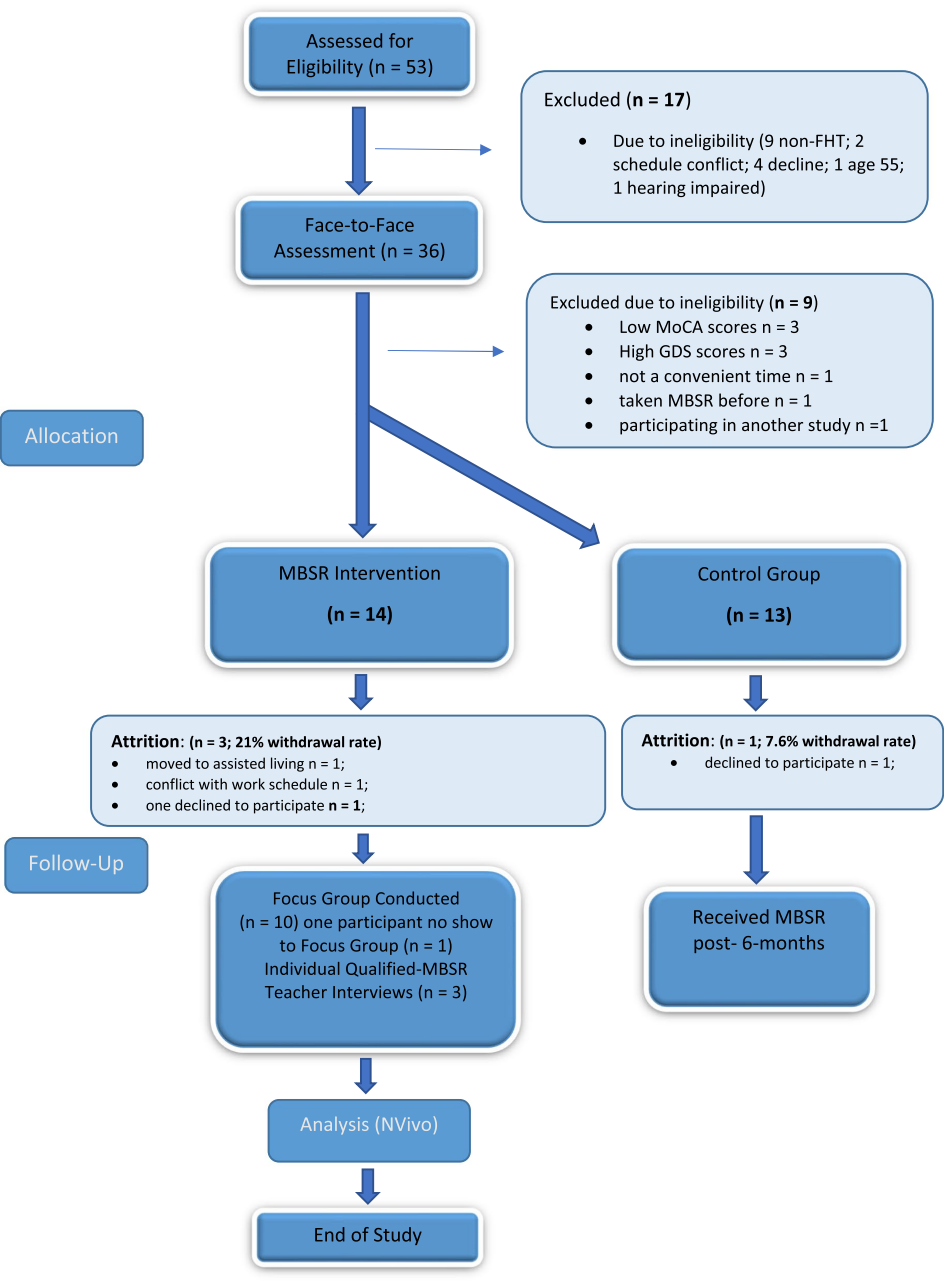
Consort flow diagram

From April to August 2019 (4.5 months), 53 patients from the FHT expressed interest in participating in the study, and were screened over the telephone by the PI of whom 36 were identified for a follow-up face-to-face screening (see Consort chart: Fig. 1 recruitment and retention of participants); 27 participants were ultimately selected and randomized, 14 to MBSR and 13 to the control group. Of note, over half of the recruitment occurred in July, the month before starting the program. (See Table 4 for recruitment data). The recruitment rate of 27 participants was 90% of our targeted recruitment rate of 30 plus participants. Based on the recruitment period of four and a half months, our study’s recruitment rate averaged about six randomized participants per month. The enrollment rate or willingness of participants to be recruited was thus 27/36, or 75%.

**Table 4:** Recruitment rates

*Recruitment Rates*	April	May	June	July	August	Total
*(April–August 2019)*	**0**	**13**	**9**	**24**	**7**	**53**
	0%	24.5%	17.0%	45.3%	13.2%	100%
*Randomized*	**0**	**5**	**6**	**14**	**2**	**27**
	0%	18.5%	22.2%	51.9%	7.4%	100%

2. *Retention rates*

**Fig. 2:**
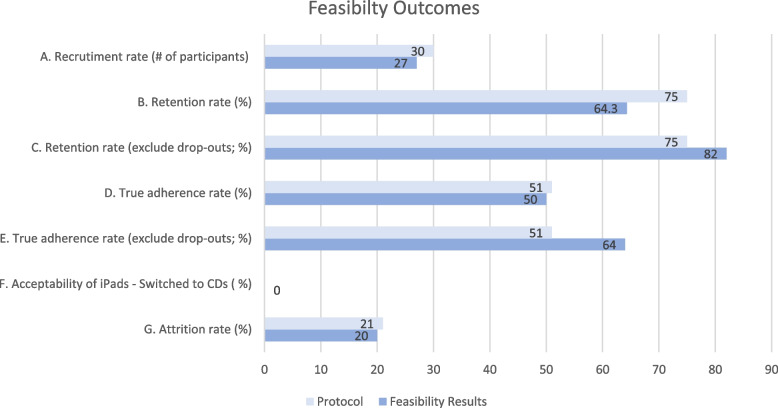
Feasibility outcomes

Following randomization, three participants assigned to the MBSR group withdrew (one dropped out before the intervention began, and another two completed parts of the program but withdrew at Week-2 or Week-3) for a withdrawal rate of 21.4% (3/14) and a moderate retention rate of 64.3% (9/14 attended ≥6 of the 10 sessions, including the All-Day and Orientation). However, excluding those three participants who withdrew early in the MBSR program, the retention rate was 81.8% (9/11 who attended ≥ 6 of the 10 weekly sessions, including the All-Day and Orientation). See Fig. 2. Therefore, 78.5% (11/14) of participants completed the program to its entirety, regardless of the number of sessions attended. In addition, 100% of participants returned the tablets or CDs and self-reported home diaries at Week-13 follow-up.

3. *Adherence rates data*
  (i) Adherence rates (tablets only; *n* = 10): five out of 10 or 50% of participants using tablets met the criteria of 39 logins (3 × 13 weeks), while four out of 10 or 40% met both the 39 logins criteria and the formal home practice criteria of ≥19.5 hrs (1.5 hrs × 13 weeks; 1170 mins). Thus, the number of participants who met the criteria for both the 39 logins and the formal home practices of ≥19.5 hrs (≥ 1170 mins) was (4/10) or 40%, a low adherence rate. Excluding those who withdrew, the adherence would have been four out of eight or 50%.
  (ii) True adherence rates (tablets and CDs; *n* = 14): seven out of 14 participants achieved both attendance cut-off (attending ≥6 sessions) and home practice (formal *and* informal practice) completion cut-off at 19.5 hrs (1170 mins) for the whole duration of the study, giving a 50% true adherence rate which is considered to be low, but excluding participants who withdrew early on in the program, the true adherence rate would have been a moderately successful 63.6% (7/11) rate overall [49]. Of note, one participant with Wi-Fi access preferred using CDs rather than tablet for formal home practice.
  (iii) Duration of formal home practice: For the 13 weeks of the study, the eight participants using tablets only practiced for 1.60 hrs or 96.4 minutes (SD = 61.1) a week (e.g., 16 mins per day, 6 days per week) on average after class. In comparison, we requested the same participants (*n* = 8) to log their formal home practice data using pen and paper. It was determined that those participants (using tablets) practiced for 131.6 mins (SD = 68.8) a week for 13 weeks (e.g., 22 mins per day, 6 days per week) after class. The discrepancy between participants logging their home practice using the tablet technology versus pen and paper documentation resulted in inflation by approximately 6 minutes/day or 27%, demonstrating an acceptable discrepancy.

#### Objectives 1(b): acceptability of technology

(i) Field notes by qualified-MBSR teachers noted that in terms of participation with technology, it was necessary to familiarize participants with the tablets and/or provide any technical support. Technical support was provided before or after the first few sessions. Two participants telephoned the PI for basic support to access the App’s home practices; however, little to no technical support was required towards the end of the program. Furthermore, technology use did not affect participating in the group. The qualified-MBSR teachers noted that most if not all participants were engaged, involved in group discussions, came prepared and were on time.
(ii) No participants who used tablets switched to low technology; for example, those who started using tablets for home practice continued doing so until the study’s end.
(iii) The results of the focus group at the end of the MBSR program are outlined in Table 5. Theme 3, *connecting to the world* highlighted the acceptability of tablet use.

**Table 5:** Major themes and sub-themes

Themes and Subthemes	Sample Quotes
***Theme 1: Unique Features of Primary Care***	
Trust and feeling safe with staff and institution	(P#11, age 61) “I trust this setting. I trust the people [staff] here.” OT #3) “there was a sense of trust; trust came up a lot for people that it felt kind of safe...”
Collaborative team-based care	OT #2) “it’s beneficial to have a connection to family physicians, other members of the team, and being able to provide care in a way that there is more collaboration and more team communication between the team members … “
***Theme 2: Satisfaction and Perceived Benefits***	
Mental health benefits	(P#21, age 60), “seeing benefits in my state of relaxation and reactions to daily issues.” (P#16, age 68), “I found it was calming … makes me feel emotionally better.” (P#18, age 61), “There has been a change in my mental karma, it is now more peaceful.”
Social Support	(P#2, age 82) “.. we end up caring about each other and taking care of each other...” (OT #3), “I think the group alone provided a sense of social support, and community for people”
Personal insights on self-care	OT #1) “across the board for most people was this realization of the need for self-kindness, self-care...”
Responding differently versus reacting	(P#7, age 79), “I’m not following my former habits. I’m sick of falling into that hole... Quicker recovery. Less ruminating.”
Adoption mindfulness practice to day-to-day activities	(P#16, age 68), “I find it easier to do [breathing] whenever I am waiting for someone or something”. (P#27, age 60), “I loved the walking one. I never thought about walking being as complex and difficult as it is.”
An alternative to medication(s)	(P#27, age 60), “I think eventually this will make some of my other pills go away.”
More than memory, other benefits	(P#11, age 61), “..This class teaches you more than memory. This is kind of like a change … on how to live your life. And it came for me in a very opportune time.”
***Theme 3: Connecting to the World***	
Acceptability of technology	(P#16, age 68), “I’m not afraid of technology.” (P#23, age 74), “I am grateful for the opportunity to learn to use the iPad.” (P#11, age 61), “I think seniors are natural to technology actually.”
Portability of technology	(P#27, age 60), “I found that it was so portable … “ OT #2) “It’s portable- this particular size of the device … [and] … very accessible at the touch of a button.”
Minimum barriers around technology	(P#23, age 74), “I was away up in a trailer for a while. And you know the CDs were helpful there, cause I didn’t have the Wi-Fi.”
The importance of support in place for technology	(P#2, age 82), “But there was also help – I had – a problem.. and it got fixed – you had a problem, and it got fixed. Mine got stuck in a place where I couldn’t figure it out. Well, actually a couple of times I had to call up [PI] and say, ‘What’s going on?’“ (P#23, age 74), “I don’t use that much technology, so using the iPad was new for me. And I had some difficulties at the beginning..” (OT #2), “a lot of questions- they were very simple questions … it was pretty much just how to use the app or how to log into the internet.”

### Qualitative findings

Three significant themes emerged from the focus group and occupational therapists’ interviews i) The unique features of primary care, ii) Satisfaction and perceived benefits of MBSR and iii) Connecting with the world. MBSR participants are identified by participant identification number (P#) and age (e.g., P#11, age 61), while the three occupational therapists are represented as OTs #1, #2 or #3.

#### Theme 1: acceptability/unique features of primary care

Many participants expressed how they trusted and felt safe with FHT staff and the institution. The MBSR teachers also noticed the theme of trust and safety. Participants with SCD or MCI reported that they “felt safe” or “very safe,” as they had pre-established relationships with other team members and their team physician. In an interprofessional primary care setting with many different providers, patients can access a wide range of programming and feel a sense of trust in these programs. As described by one participant (P#13, age 68), “I was recommended to this program by my doctor; she listened to me and recommended [this program],” demonstrating the collaborative nature of primary care. Patients can also self-refer themselves as services are advertised in the FHT waiting room, e.g., television, flyers etc. One participant (P#7, age 79) said, “I saw these flyers on the third floor … and then I went, ‘Oh my god, this is exactly what I need because … I thought well maybe this will help” which describes a growing potential need within this population in primary care, in which patients can self-refer in an interprofessional primary care setting. Sample quotations are found in Table 5.

#### Theme 2: satisfaction and perceived benefits of MBSR

Participants described their perceived benefits and satisfaction based on the following subthemes: i.) mental health benefits, ii.) social support iii.) personal insights on self-care, iv.) responding differently vs. reacting, v.) adoption of mindfulness practice to everyday activities, vi.) alternative to medication(s), and vii.) more than memory.

Participants described their perceived value and satisfaction with the MBSR program by describing high satisfaction with mental health benefits, reporting “feeling more relaxed,” “emotionally better,” “peaceful,” “calm,” and “getting in touch with one’s emotions.” There was also the added benefit of social support, which decreased social isolation or loneliness, especially for those living with SCD or MCI. One participant noted the group “allowed us to connect (P#16, age 68)”, and another that, “it keeps me connected throughout the week (P#23, age 74)”. The MBSR group also provided personal insights into “self-care”. It was noted by OT#1 that, “for most people was this realization the need for self-kindness, self-care, self-compassion (OT#1)”. One participant realized that, with ageing and living with cognitive issues, “ … you have to take different ways … to take care of yourself now (P#16, age 68)”, and another participant stated that, “self-acceptance is self-care … and perhaps most of us have not really thought about it that way before (P#2, age 82).”

A subtheme emerged was learning to have a different approach to stress by responding differently versus reacting. Most participants developed an increase in awareness and paying attention to stress reactivity which promotes recovery from stressful situations faster and with less rumination. As mentioned by a participant, “I’m not following my former habits. I’m sick of falling into that hole. Quicker recovery. Less ruminating”. (P#7, age 79).“ With mindfulness practice, there is a component of incorporating it into everyday activities noted by a participant who stated, “I find it easier to do [breathing] whenever I am waiting for someone or something (P#16, age 68)“ by using opportunities in the day to do a quick mindfulness practice. Another participant would make a grocery list, but forgets it at home and said, “I focus on what I’m writing … I don’t have the list with me, so it’s making me think more and be aware of myself, listening … and developing more strategies (P#13, age 68)“. Participants also spoke about how mindfulness can broadly support their health and as an alternative to medications. One participant mentioned that, “ … I’ve had issues with my back, and I was taking all these medications, and they were making me feel worse! (P#13, age 68)“ and another participant said similar sentiment by saying, “I think eventually this will make some of my other pills go away (P#27, age 60).”

Lastly, participants stated that the program was more than memory as it gave them tools to approach life from a different perspective. As mentioned by a participant, “this class teaches you more than memory. This is kind of like a change … on how to live your life. And it came for me in (sic) a very opportune time (P#16, age 68)”. The program is about learning to pay attention, and one participant stated, “my listening skills are much better … [mindfulness practice] makes me focus … (P#13, age 68)” noticing an improvement in listening and focusing, which is a cognitive benefit. To summarize, an older participant expressed intense appreciation for the group and said, “ … I think we need more programs like this. (P#8, age 86)”.

#### Theme 3: acceptability of technology, connecting with the world

Participants and occupational therapists described using iPads for home practice as i.) acceptable, ii.) portable, iii.) involving minimal barriers and iv.) demonstrating importance of supports in learning how to use technology in the MBSR program.

OT#1 noticed participants’ enthusiasm and receptiveness to technology, stating, “They’re in love with technology! They’re open to it … “. Many participants echoed similar sentiments and were “grateful for the opportunity to learn to use the iPad (P#23, age 74)”. Secondly, OT#2 described computer tablets as, as portable and therefore accessible for home practice because of their “particular size of the device … [and] … very accessible at the touch of a button”. Participants noted that they could use their headphones and do a practice in any room of their home. The only two minor barriers from participants’ perspectives on technology use included the learning curve and access to Wi-Fi when outside their homes. Lastly, it is important to ensure that assistance is available and integral to support participants’ success in learning and using technology independently. No participants raised any challenges about the program or identified any barriers to the acceptability of the technology.

## Discussion

In this convergent mixed-methods, single-blind, pilot RCT we demonstrated that a 9-week occupational therapy-led MBSR program was feasible for practice among community-dwelling, older adult primary care patients living with SCD or MCI. However, based on the lower-than-expected rates of recruitment, retention, and adherence, our study as designed did not meet the feasibility bench marks that were set. However, with minor modifications to the design, including changing how participants who drop-out are analyzed, extending recruitment, adding multiple sites, this intervention would be well suited to further study using a full-scale RCT.

Following our research protocol, we used last observation carried forward (LOCF) method to manage drop-outs. As such, since three participants withdrew early from the study for reasons unrelated to the MBSR program, the retention rate was below our specified 75–80% benchmark because a small number of drop-outs impacted our results based on the LOCF approach used in our protocol. Excluding participants who withdrew, the study’s retention rate would have been higher. Similarly, the study had low adherence and true adherence rates but, excluding participants who withdrew early, the true adherence rate would have been moderate, compared to other mindfulness adherence studies [50]. Additionally, participants who remained in the intervention group to its entirety remained committed despite the substantial time commitment. Lastly, although our study’s feasibility outcomes were lower than expected, a high number of participants completed the entire MBSR program, and we had a high follow-up rate in an FHT. Based on our findings, it is recommended that a future trial may consider using an alternative LOCF method, as we noted that drop-outs significantly impacted our rates of retention and adherence. Second, the recruitment period (4.5 months) should be longer or the MBSR program offered at multiple primary care sites to obtain a larger sample size.

As the study proceeded, we made two changes to the original study protocol. The first change was to modify the inclusion criteria to clarify how MCI was determined. An MCI diagnosis in the EMR would meet the study’s eligibility, but in cases where there was no MCI confirmation in the EMR, but a participant’s MoCA score was ≤23, we used Petersen et al.’s (2014) MCI criteria for eligibility [4]. Second, we changed the retention rate, from completing 6 ≥ of the 9 sessions to 6 ≥ of the 10 sessions to capture the orientation session, resulting in a moderate retention rate of 64.3% (9/14), although it was below our specified 75–80% benchmark. If the orientation session was excluded as per our original study protocol, it would have been a lower retention rate of 50% (7/14 attended ≥6 of the 9 sessions). The decision to include the orientation session in our retention rate was because it was similar in structure to the other weekly sessions (provided in a three-hour group format). In contrast, other MBSR studies conducted a one-hour orientation session individually and right after the randomization process or did not provide a description of their orientation session [51, 52].

Our findings are consistent with the growing body of literature on the benefits of mindfulness-based interventions in primary care [53]. However, we only achieved 90% of our targeted 30 plus participants. To achieve our recruitment target, the recruitment period would need to be extended from 4.5 months to at least six months or to include multiple primary care sites to achieve a larger sample size and the recruitment rate.

Our study also supports other recent RCT findings that MBSR is feasible for practice and is an optimal early intervention for the SCD population [33, 54, 55]. Our analysis also demonstrated that using technology such as computer tablets for program delivery was feasible and highly acceptable to our participants living with cognitive complaints. Based on our results, embedding the use of this technology in a primary care setting such as an FHT would be viable. As the numbers of community-dwelling older adults with SCD or MCI increase in the coming years, strategies that support their functional and psychosocial needs, especially from a non-pharmacological perspective, are critical. There is currently limited information on the types of healthcare services provided to older adults with SCD or MCI in interprofessional primary care settings. For example, a national survey by Donnelly et al. (2016) has shown that 57% of occupational therapists’ caseload in primary care involves providing support to older adults. Health promotion and prevention-related activities (75%) followed by group-based interventions (14%) were the most frequently described interventions in this setting [56], and this aligns well with our study noting the benefits of such an occupational therapy-led MBSR group. A study by Mirza et al. (2020) determined that an occupational therapy group-based intervention in primary care was feasible, acceptable and highly satisfying to older adults living with chronic conditions [57] which further supports our findings. These findings are consistent with many other studies showing that an occupational therapist-led MBSR is feasible for practice among participants with MCI or mild dementia [58], as demonstrated by the qualitative data of the focus group and noting low attrition; high completion; and a 100% follow-up. Further, excluding those three participants who withdrew, the results demonstrate moderate true adherence rates along with high retention rates.

Our qualitative investigation found that MBSR program supports individuals with cognitive complaints such as SCD or MCI through a broad range of positive effects, such as mental health benefits of group process including “social support” and “feeling safe.” These findings were consistent with a mixed-methods study by Berk et al. (2018), which found similar themes in a group process among middle-aged and older adults with memory complaints while attending an MBSR program [59]. Other studies have noted a reduction in worrying about memory complaints [59] and increased memory self-efficacy following MBSR [33]. Also, our study participants gained more insights into how to manage their everyday lives. Perhaps future, more extensive trials may consider evaluating the impact of incorporating MBSR programs within this population’s everyday life participation. Consistent with the literature, there were no noted adverse events [60, 61].

Embedding MBSR in a primary care setting may effectively meet the complex and unique needs of this population by supporting and managing their health care needs, including psychosocial support. The unique feature of collaborative practice in primary care is communication between providers. With this collaborative practice, it is developing a partnership between interprofessional primary care providers and patients to negotiate and navigate their health care needs. This results in patients establishing a more personal connection with interprofessional primary care providers on the team. Feelings of trust and safety are pre-established elements that can be leveraged for adopting such a program in an interprofessional primary care setting.

Participants with SCD or MCI were open and receptive to and were capable of learning how to use computer tablets. This receptiveness was demonstrated in Wahbeh et al.’s study (2016), where they found a high recruitment rate (75%) and acceptability of an internet-based mindfulness meditation intervention for cognition and mood among older adults. However, Wahbeh’s et al. (2016) study contradicts ours slightly; they stated that it was too difficult for older adults to use iPads or iPod touch and they had to be switched over to participants’ desktop and laptop computers if they owned them. The Wahbeh et al. (2016) study is 6 years old (and the data is potentially older) which could explain the discrepancies as older adults are becoming more and more proficient with using technology. Wahbeh et al. (2016) also recommended that older adults need some basic knowledge of computers and how they work and to have their own computers to access online programs. Only 38% (3/8) of our study participants who used the tablets had minor issues accessing the App or Wi-Fi access. However, once support was provided, they independently used the tablets thereafter. Providing technological support in the initial phase of the study was crucial. The literature supports the importance of providing assistance with technology and that technology-based instructions require a high structure environment along with ensuring helpful resources are readily available [62] to ensure successful use of technology. It could be argued that participants who had Wi-Fi in their homes or who own smartphones were more receptive to and comfortable with using technology as demonstrated by eight out of 10 or 80% of participants choosing tablets. The provision of access to Wi-Fi in our study for tablet use provided valuable insight into the acceptability of technology for MBSR program delivery and data collection. However, we found that being flexible and inclusive of participants’ comfort levels by also offering a CD option for home practice is an important factor to ensure that participants fully participate in the MBSR program. Technology-based mindfulness programs are on the horizon for older adults. Choo et al. (2018) reported on the potential use of a smartphone App to deliver mindfulness interventions to older Asian adults at risk for suicide [63]. In another study, Ungar et al. (2019) used an online mindfulness program that was greatly beneficial to lonely older adults [64]. The literature exemplifies a growing use of technology to support older adults to “age-in-place.” The qualitative data from our focus group and interviews with the occupational therapists, validates embedding technology in an interprofessional primary care setting. Future studies could explore the use of technology in primary care program delivery and research.

A recent systematic review and meta-analysis of 43 interventional mindfulness studies by Parsons et al. (2017) found that average home practice was 180 minutes (SD = 43) a week (e.g., 30 minutes per day, six days a week) among participants (general adults). In our study, based on the direct measure (i.e., tablets), participants practiced for 96.4 minutes/week (SD = 61.1) at home (e.g., 16 minutes per day on average). However, based on the subjective self-report measure (i.e., home practice diary), participants practiced for 131.6 mins (SD = 68.8) a week for 13 weeks (e.g., 22 min per day, 6 days per week on average) outside of class time. This demonstrates approximately 6 minutes or about 27% inflation in subjective self-report (i.e., home practice diaries). This study is consistent with the literature reporting a discrepancy between direct versus subjective self-reported measures [65]. Nevertheless, since there only appears to be a 6-minute discrepancy, it could be argued that participants in our study were acceptable accuracy with their self-reporting. In a study by Wahbeh et al. (2016), iPad/iPod was used, but participants were not asked to report their analysis of home practice frequency and duration of practice per day for their mindfulness-based internet intervention. Thus, to the author’s knowledge, this is the first study to describe the use of direct measures using tablets to collect formal home practice versus subjective self-report measures (i.e., home practice dairy) in an MBSR program. Secondly, to our knowledge, there is no home practice data in the literature of older adults living with cognitive complaints for comparison at present.

Direct measure (i.e., tablet) does come with limitations and consideration of using subjective self-reporting may be worthwhile to include in the overall evaluation of home practice data. The adherence rate of completing formal home meditation practice on tablets was 40% (4/10), as opposed to the higher true adherence rate which was 50% that also captured informal homework practices or behavioural change. Informal practices such as deliberately adopting attention to everyday activities and noticing the experience (e.g., walking, eating, washing dishes, etc.) are important. The lower adherence rate was accounted for by situations when participants could not capture home practice data. For example, one participant did not have Wi-Fi access when she was at her cottage, while other participants travelled during the summer months without their tablets for fear of damaging or losing them. Self-reported home practice dairy sheets were, therefore, necessary to determine the true adherence rate. The true adherence rate is essential since attending weekly MBSR sessions alone is insufficient. Excluding participants that withdrew, the higher true adherence rate positively reflects participants’ high level of engagement and adherence to the MBSR program, and it also validates their overall practice quality [66]. Parsons et al. (2017) reported that participants’ level of engagement with home practice is essential, and there was a statistically significant association between participants’ home practice and the impact on health outcomes [67].

Some minor barriers in this tablet-based practice included lack of reliable or general Wi-Fi access, which was similarly noted by Schepens et al. (2018), where unreliable Wi-Fi made it difficult at times in carrying out the intervention [68]. Only two participants in the intervention group did not have home Wi-Fi and needed CDs for their home practice. However, one participant with Wi-Fi access preferred using CDs rather than tablet for formal home practice. Those participants who used tablets required help during the first few sessions, but thereafter, all could conduct their home practices independently. Research on technology acceptance by community-dwelling older adults has emerged. Our study showed that older adults living with SCD or MCI are highly receptive to learning how to use technology, and future group intervention programs in interprofessional primary care settings may also incorporate tablet use.

### Strengths and limitations

To our knowledge, this is the first study that examined the feasibility and acceptability of technology use in an occupational therapist-led MBSR program for older adults living with cognitive complaints in a team-based primary care setting. The small sample size was drawn from one urban primary care site, and our participants were mainly of a higher socioeconomic status, and results may be different from a more socially disadvantaged group of older adults. The norm is to have only two MBSR teachers but having two additional MBSR teachers in the study to support participants’ technological issues may have impacted their experience and may not be feasible in a typical setting. In addition, only five participants (5/14) met the MCI criteria as two had a confirmed diagnosis of MCI, while the other three did not, but met the MCI eligibility based on Petersen’s et al.’s (2016) criteria. Thus, this small number of participants with MCI may limit the findings of feasibility and acceptability of technology use for the MCI population. Furthermore, our retention rates and true adherence rates were higher when we excluded the three participants who left the study. Thus, a future randomized clinical trial should consider (i) an alternative design method if LOCF to manage drop-out and (ii) using multiple primary care sites to achieve a larger sample size to include participants from diverse socioeconomic backgrounds and to enhance the evaluation of the impact of changes in everyday life participation with MBSR.

## Conclusions

Based on our lower than expected rates of recruitment, retention, and adherence we found that our study did not meet the feasibility benchmarks as proposed in the current research design. However, this study highlighted that MBSR is feasible for practice, in an older adult population with early cognitive impairment in an interprofessional primary care setting. A future RCT is recommended with changes to the design to account for the findings of this study. A future trial may consider using an alternative LOCF method, we noted that drop-outs significantly impacted our rates of retention and adherence. Second, the recruitment period should extend beyond 4.5 months or the program offered at multiple primary care sites to achieve a larger sample size and the desired recruitment rate.

Our participants reported on their satisfaction and perceived benefits from the qualitative data and there is much promise for this intervention as it is well aligned with the philosophies of primary care and team-based models. The study’s findings uphold the acceptability and portability of technology and illustrate those persons with SCD or MCI are receptive to learning and using computer tablets. Thus, future group MBSR programs in primary care may adopt technology use with this population. This study adds to the sparse literature on types of health care delivery in an interprofessional primary care setting and the vital contribution of occupational therapy to better support individuals with complex health conditions compared with physician care alone. This study will inform future research for evidence-based, occupational therapy-focused interventions on how best to deliver care to older adults with cognitive complaints such as SCD or MCI in an interprofessional primary care setting.

## Supplementary Information

**Additional file 1: Appendix 1.** Summary table of retention, login frequency, duration of practice, adherence and true adherence rate. **Appendix 2.** Retention Data 9/14 participants or 64.2%. **Appendix 3.** Login Frequency with Tablets 5/10 or 50% login frequency. **Appendix 4.** Login Duration of Formal Home Practice on Tablets.

## Data Availability

The datasets collected and analyzed during the current study are not publicly available to maintain privacy and confidentiality of our participants enrolled in this trial as per policy and health care system regulations of the trial site. The datasets used and analyzed during the current study is available from the corresponding author on reasonable request.
