# Supplementary material for: Mindfulness-based stress reduction for community-dwelling older adults with subjective cognitive decline (SCD) and mild cognitive impairment (MCI) in primary care: a mixed-methods feasibility randomized control trial
Source: BMC Prim Care. 2023 Feb 9;24:44. doi: 10.1186/s12875-023-02002-y (PMC9912594; doi:10.1186/s12875-023-02002-y)
Supplement: Supplementary file 1 — Additional file 1: Appendix 1. Summary table of retention, login frequency, duration of practice, adherence and true adherence rate. Appendix 2. Retention Data 9/14 participants or 64.2%. Appendix 3. Login Frequency with Tablets 5/10 or 50% login frequency. Appendix 4. Login Duration of Formal Home Practice on Tablets. [file 12875_2023_2002_MOESM1_ESM.docx]

Appendix 1 – Summary table of retention, login frequency, duration of practice, adherence and true adherence rate

|  |  | |  | |  |  | |  | | | |  |
| --- | --- | --- | --- | --- | --- | --- | --- | --- | --- | --- | --- | --- |
| N = 14 | Participants | (iPad or CD) | | Retention rate  (completion of 6 $\geq$ sessions) | | | Login Frequency (3 logins/per week ≥ 39 logins)  iPad ONLY | | Total  Hours of 19.5 hrs or more  iPad and CDs  Formal Only | Total Hours of 19.5 hrs or more  iPad and CDs  Combined formal and informal practice 19.5 hrs (All Participants) | *Adherence rate*  (with 39-logins and only formal practice ≥19.5 hrs)  iPad ONLY | *True adherence rate*  (with completion of ≥6 sessions combined with both formal and informal practice ≥ 19 hrs)  iPad and CDs – All Participants |
| 1 | Participant 2 | iPad | | Yes | | | No | | No | Yes | No | Achieved (1/7) |
| 2 | Participant 7 | iPad | | Yes | | | Yes | | Yes | Yes | Achieved (1/4) | Achieved (2/7) |
| 3 | Participant 8 | CD | | Yes | | | No (CD) | | No | No | No | No |
| 4 | Participant 11 | iPad | | Yes | | | Yes | | Yes | Yes | Achieved (2/4) | Achieved (3/7) |
| 5 | Participant 12 | CD | | No | | | No (CD) | | No | No | No | No |
| 6 | Participant 13 | iPad | | Yes | | | No | | No | No | No | No |
| 7 | Participant 16 | CD | | Yes | | | No (CD) | | Yes | Yes | No | Achieved (4/7) |
| 8 | Participant 18 | iPad | | No | | | Yes | | No | No | No | No |
| 9 | Participant 21 | iPad | | Yes | | | Yes | | Yes | Yes | Achieved (3/4) | Achieved (5/7) |
| 10 | Participant 23 | iPad | | Yes | | | Yes | | Yes | Yes | Achieved (4/4) | Achieved (6/7) |
| 11 | Participant 27 | iPad | | Yes | | | No | | No | Yes | No | Achieved (7/7) |
| 12 | Participant 4 | iPad | | Withdrew | | |  | |  |  |  |  |
| 13 | Participant 17 | iPad | | Withdrew | | |  | |  |  |  |  |
| 14 | Participant 25 | No Show | | Withdrew | | |  | |  |  |  |  |
|  | Summary  3/14 = 21% attrition  11/14 = 78.5% remained | 10 iPads  3 CDs  1 No Show | | 9/14 = 64.3% (moderately successful)  Exclude those withdrew: 9/11 = 81.8% - high retention rate | | | 5/10 = 50%; login frequency  Exclude those withdrew: 5/11 = 5/8= 62.5% | | 5/14 = 35.7%  Exclude those withdrew: 5/11 = 45.4% | 7/14 = 50%  Exclude those withdrew: 7/11 = 63.6 | 4/10 = 40%; adherence rate (iPad only)  Exclude those withdrew: 4/8 = 50% | 7/14 = 50%; True adherence rate (iPad and CDs); Exclude those withdrew: 7/11 = 64%  NB: 6/10= 60%; true adherence rate (iPad ONLY); Exclude those withdrew: 6/8 = 75% |

Appendix 2 – Retention Data 9/14 participants or 64.2%,

(Excluding those who withdrew 9/11 participants or 82% retention rate)

| n | Attendance | Orientation | Week 1 | Week 2 | Week 3 | Week 4 | Week 5 | Week 6 | Week 7 (All Day) | Week 8 | Week 9 | Missed Days | Attendance Rate (10 sessions) |  |
| --- | --- | --- | --- | --- | --- | --- | --- | --- | --- | --- | --- | --- | --- | --- |
| 1 | Participant 2 | yes | no | yes | yes | yes | yes | no | yes | yes | yes | 2 of 10 | 80% | Achieved (1/9) |
| 2 | Participant 4 | yes | yes | yes | Withdrew | |  |  |  |  |  | 7 of 10 | 30% |  |
| 3 | Participant 7 | yes | yes | yes | no | yes | yes | yes | yes | yes | yes | 1 of 10 | 90% | Achieved (2/9) |
| 4 | Participant 8 | yes | yes | yes | yes | no | yes | yes | yes | yes | yes | 1 of 10 | 90% | Achieved (3/9) |
| 5 | Participant 11 | yes | yes | no | yes | yes | yes | yes | no | yes | yes | 2 of 10 | 80% | Achieved (4/9) |
| 6 | Participant 12 | yes | no | no | no | yes | yes | no | yes | yes | no | 5 of 10 | 50% |  |
| 7 | Participant 13 | yes | yes | yes | no | no | yes | yes | no | no | yes | 4 of 10 | 60% | Achieved (5/9) |
| 8 | Participant 16 | yes | yes | yes | yes | no | yes | yes | yes | yes | yes | 1 of 10 | 90% | Achieved (6/9) |
| 9 | Participant 17 | yes | yes | Withdrew | |  |  |  |  |  |  | 2 of 10 | 20% |  |
| 10 | Participant 18 | yes | no | no | yes | yes | yes | no | no | no | no | 6 of 10 | 40% |  |
| 11 | Participant 21 | yes | yes | yes | yes | yes | yes | yes | yes | yes | yes | 0 of 9 | 100% | Achieved (7/9) |
| 12 | Participant 23 | yes | no | yes | yes | yes | yes | yes | yes | yes | yes | 1 of 10 | 90% | Achieved (8/9) |
| 13 | Participant 25 | Withdrew | |  |  |  |  |  |  |  |  | 0 of 10 | 0% |  |
| 14 | Participant 27 | yes | no | yes | no | yes | no | yes | no | yes | yes | 4 of 10 | 60% | Achieved (9/9) |

Appendix 3 – Login Frequency with Tablets 5/10 or 50% login frequency

(Excluding those who withdrew, 5/8 or 62.5% login frequency)

|  | Computer Tablet (iPad) Homework Logins Frequency | | | | | | | | | | | | | | | |
| --- | --- | --- | --- | --- | --- | --- | --- | --- | --- | --- | --- | --- | --- | --- | --- | --- |
|  | N = 10 (for those who only used iPads) | Week 1 | Week 2 | Week 3 | Week 4 | Week 5 | Week 6 | Week 7  All-Day | Week 8 | Week 9  Post-MBSR | Week 10 | Week 11 | Week 12 | Week 13  F/U | Total Logins | Goal Achieved  3 logins/per week $\geq$ 39 logins |
| 1 | Participant 2 | 2 | 4 | 3 | 2 | 1 | 0 | 1 | 2 | 0 | 0 | 0 | 0 | 0 | 15 |  |
| 2 | Participant 7 | 5 | 6 | 6 | 5 | 3 | 8 | 5 | 7 | 5 | 7 | 6 | 7 | 7 | 77 | Achieved 1/5 |
| 3 | Participant 11 | 5 | 5 | 4 | 5 | 4 | 6 | 2 | 2 | 2 | 3 | 4 | 1 | 0 | 43 | Achieved 2/5 |
| 4 | Participant 13 | 5 | 3 | 2 | 2 | 3 | 1 | 0 | 0 | 0 | 0 | 0 | 0 | 0 | 16 |  |
| 5 | Participant 18 | 0 | 0 | 5 | 9 | 1 | 1 | 6 | 5 | 1 | 0 | 1 | 10 | 2 | 41 | Achieved 3/5 |
| 6 | Participant 21 | 5 | 6 | 10 | 6 | 11 | 6 | 5 | 3 | 2 | 1 | 0 | 1 | 0 | 56 | Achieved 4/5 |
| 7 | Participant 23 | 4 | 6 | 0 | 7 | 6 | 6 | 5 | 9 | 5 | 7 | 7 | 7 | 2 | 71 | Achieved 5/5 |
| 8 | Participant 27 | 3 | 7 | 6 | 1 | 2 | 4 | 0 | 0 | 0 | 0 | 0 | 0 | 0 | 23 |  |
| 9 | Participant 4 | withdrew |  |  |  |  |  |  |  |  |  |  |  |  |  |  |
| 10 | Participant 17 | withdrew |  |  |  |  |  |  |  |  |  |  |  |  |  |  |

Appendix 4 – Login Duration of Formal Home Practice on Tablets

4/10 participants or 40%; (excluding those who withdrew 4/8 participants or 50% achieved $\geq$ 19.5 hrs adherence rate for iPad)

|  | iPad Homework Duration | |  |  |  |  |  |  |  |  |  |  |  |  |  |  |  |
| --- | --- | --- | --- | --- | --- | --- | --- | --- | --- | --- | --- | --- | --- | --- | --- | --- | --- |
|  | N = 10 (for those who only used iPads) | Week 1 | Week 2 | Week 3 | Week 4 | Week 5 | Week 6 | Week 7  All-Day | Week 8 | Week 9  Post-MBSR | Week 10 | Week 11 | Week 12 | Week 13  F/U | Total (Minutes) | Total (Hours) | Goal Met  $\geq$ 19.5 hrs (1,170 mins) |
| 1 | Participant 2 | 69.6 | 139.8 | 90.6 | 63.6 | 16.8 | 0 | 20.4 | 21.6 | 0 | 0 | 0 | 0 | 0 | 422.4 | 7.04 |  |
| 2 | Participant 7 | 154.8 | 209.4 | 183 | 144 | 86.4 | 202.8 | 155.4 | 267.6 | 184.2 | 249.6 | 204.6 | 253.8 | 204.6 | 2,500.2 | 41.67 | Achieved (1/4) |
| 3 | Participant 11 | 186.6 | 186.6 | 135 | 145.2 | 125.4 | 189.6 | 61.8 | 61.8 | 67.2 | 86.4 | 132 | 16.8 | 0 | 1,394.4 | 23.24 | Achieved (2/4) |
| 4 | Participant 13 | 141.6 | 93 | 63.6 | 67.2 | 88.2 | 22.8 | 22.8 | 0 | 0 | 0 | 0 | 0 | 0 | 499.2 | 8.32 |  |
| 5 | Participant 18 | 0 | 0 | 143.18 | 134.11 | 3.34 | 40.15 | 154.2 | 59.17 | 3.33 | 0.05 | 38.12 | 193.26 | 9.52 | 778.43 | 12.9738333 |  |
| 6 | Participant 21 | 240.6 | 204.6 | 138.6 | 136.8 | 204.6 | 142.8 | 135.6 | 90.6 | 34 | 34 | 0 | 34 | 0 | 1,396.2 | 23.27 | Achieved (3/4) |
| 7 | Participant 23 | 142.2 | 126 | 1 | 201.6 | 209.4 | 191.4 | 150.6 | 261.6 | 180.6 | 247.2 | 256.2 | 256.2 | 69.6 | 2,293.6 | 38.23 | Achieved (4/4) |
| 8 | Participant 27 | 93.6 | 256.2 | 209.4 | 38 | 70.2 | 73.8 | 0 | 0 | 1 | 0 | 0 | 0 | 0 | 742.2 | 12.37 |  |
| 9 | Participant 4 | withdrew |  |  |  |  |  |  |  |  |  |  |  |  |  |  |  |
| 10 | Participant 17 | withdrew |  |  |  |  |  |  |  |  |  |  |  |  |  |  |  |

Therefore, adherence rate (tablets only): 40% or participants achieved both 39 logins and 19.5 hrs of home practice.
